# An Individualized Training Program for PE Teachers Based on Self-Determination Theory as a Way to Improve Students’ Psychosocial Health: A Study Protocol

**DOI:** 10.3390/ijerph20166604

**Published:** 2023-08-18

**Authors:** Alba González-Peño, Evelia Franco, Laura Martín-Hoz, Javier Coterón

**Affiliations:** 1 Faculty of Physical Activity and Sport Sciences—INEF, Universidad Politécnica de Madrid, C/Martín Fierro, 7, 28040 Madrid, Spain; laura.martin.hoz@alumnos.upm.es (L.M.-H.); j.coteron@upm.es (J.C.); 2 Facultad de Ciencias Humanas y Sociales, Universidad Pontificia Comillas, C/Universidad Comillas, 3–5, 28108 Madrid, Spain; efalvarez@comillas.edu

**Keywords:** motivation, circumplex approach, motivational strategies, individualization, observation

## Abstract

The interactions that take place in physical education (PE) between teachers and students have received large attention from the scientific community. However, despite the existence of different studies aiming to promote motivation among students through school interventions, there seem to be no interventions based on motivational strategies in which interventions are personalized to better fit teachers’ own characteristics on the basis of theoretical contents grounded in self-determination theory. This study aims to present a protocol intervention in the PE context based on SDT to improve teaching behaviours through an individualized and lifelong training program. This protocol is a convenience study in which PE teachers will design and implement motivational strategies to increase students’ motivation in class. The training program will take place along the intervention to allow teachers to personalize their implementation of motivational strategies according to their specific context. Data collection will be conducted before, during and after the intervention using recorded sessions (observational methodology), interviews (qualitative approach), and questionnaires (quantitative approach). The measures will assess teachers’ and students’ perceived teaching styles. This intervention program is expected to change and improve the quality of teaching behaviours, which could foster students’ psychosocial health.

## 1. Introduction

The interactions that take place in the physical education (PE) class between teachers and students have gained much attention from the research community in the last few years, since teachers’ motivational patterns have been found to be closely related to students’ behaviours [1,2]. Self-determination theory (SDT) [3,4] has been used to explain how teachers’ motivational processes fluctuate between higher and lower levels of self-determination and influence different behavioural outcomes in the PE context. According to this theory, a person can exhibit autonomous motivation, controlled motivation or amotivation depending on the level of self-determination, which refers to the extent to which behaviours are voluntary. Specifically, in the educational context, when teachers experience autonomous motivation, they can present either intrinsic motivation, related to the inherent pleasure towards teaching, or an identified regulation, related to the recognition of teaching values. Teachers can also experience controlled motivation when they feel pressure to act in a certain way, by presenting introjected regulation, related to internal pressures to avoid feeling of guilty, or external regulation, related to external pressures to conform with a demand. Finally, when teachers show amotivation, they probably have no reasons to teach.

This theory also posits that the satisfaction of the three basic psychological needs (BPN) of autonomy, competence and relatedness will lead to autonomous motivation and, in turn, to optimal psychological functioning in a person [5]. Related to teachers, they can experience autonomy satisfaction in teaching when they are able to implement their own proposals and new ideas. Perceptions of competence will occur when they feel able to manage groups. Finally, they can experience relatedness when they feel they are supported by their colleagues. However, the frustration of BPN will lead to controlled motivation and amotivation, and, consequently, will influence teachers’ well-being as well as interactions with their students [6]. Although frustration was originally understood as a lack of satisfaction of BPN, the evidence showed that did not simply reflect the perception that satisfaction is low, but that BPN are being actively threatened [7]. In this sense, previous studies have shown how teachers’ frustration can be manifested when teaching [8]. Teachers may feel their autonomy is frustrated if they are not able to decide their own methodological approaches. Teachers may perceive their competence as being undermined if they are not given the opportunity to demonstrate their teaching skills. Finally, teachers may perceive that their relatedness is being frustrated if they feel excluded by their colleagues at work.

### 1.1. The Circumplex Approach: A New Perspective to Understand PE Teaching Behaviours

The existing literature points out that need-supportive teaching behaviours will enhance students’ motivation [9]. On the other hand, need-thwarting behaviours will arise when teachers exercise power as an authority by demanding respect, ignoring students’ interests or pressuring them through their self-confidence [10]. In the last few years, it has been suggested that reducing these controlling behaviours and promoting certain motivational strategies according to BPN satisfaction [11] will foster more adaptative outcomes between students [2]. In this sense, a more fine-grained and innovative approach has emerged for a deeper understating of the interactions between teachers and students through the analysis of teachers’ behaviours in the educational context [12]. This model distinguishes four different dimensions according to the level of directiveness (located in the vertical axis) and the level of satisfaction or thwarting of BPN (located in the horizontal axis), and it does not consider each need independently, but rather establishes an overall measure. These four dimensions are characterized by the combination of high or low levels of both axes. Needs support and low directiveness result in an autonomy-supportive teaching style, whereas needs support and high directiveness result in a structured teaching style. Needs thwarting and high directiveness result in a controlling teaching style, while needs thwarting and low directiveness result in a chaotic teaching style. Within each dimension, teachers can adopt eight specific teaching styles. Related to the autonomy-supportive dimension, a participative teacher will be characterized by identifying students’ personal interests and offering choice; an attuning teacher will look for modifications to make activities more attractive and enjoyable. Related to the structure dimension, a guiding teacher may suggest individual progressions to help students to complete the activities; a clarifying teacher will be clear and transparent when communicating expectations from the lesson and students’ behaviours. Related to the controlling dimension, a demanding teacher will use powerful language to require discipline and try to change students’ thoughts or ideas; a domineering teacher will induce feelings of shame and guilt. Finally, related to the chaotic dimension, an abandoning teacher will leave students on their own; an awaiting teacher will not plan the lesson and students will have to always take the initiative.

Despite dozens of researchers addressing the study of motivational processes in physical-activity-related educational contexts, studies aiming to explore motivational outcomes applying the circumplex approach are less abundant due to the recent and innovative nature of this approach. The study carried out by Moè et al. [13] was developed in middle and high school using the circumplex approach; Vermote et al. [14] were the first one to carry out an analysis of the university context; Franco et al. [15] analysed student teachers’ perceptions of their teachers’ styles in the university context. Even though the literature is scarce, the circumplex approach seems an excellent perspective to deepen the understanding of the interactions between teachers and students that take place in PE classes. In this line, a recent study proposed a system classification to identify different teachers’ motivational behaviours related to students and consistent with SDT [11]. This classification organizes teachers’ behaviours according to the satisfaction or thwarting of student’s needs of autonomy, competence, and relatedness. A total of 57 motivational strategies that could occur in a PE class were proposed. Once again, due to the innovative nature of this research, there are no previous studies that have used this system classification to address how teachers interact with their students in real-life practice. However, a recent study presented an approach to integrate the motivational strategies from Ahmadi et al. [11] and the different teaching styles from Aelterman et al. [12,16] in the PE context, establishing a relationship between each strategy and the four big dimensions of the autonomy-supportive, structure, control and chaotic teaching styles [17]. This interesting proposal would be the first attempt to specify which motivational strategies are related to each teacher profile.

### 1.2. SDT-Based School Interventions

Despite the existence of previous studies on SDT-based school interventions that have found positive outcomes between students when their teachers create a motivational climate and support pupils’ motivational processes [18,19], the focus of this type of study has always relied on student outcomes. However, the interactions that take place in PE classes involve both the teacher and students, and the influence that each has on the other has been evidenced [1]. In this regard, it seems that not only students’ outcomes in school interventions should have attention but teachers’ training programs, and how teaching behaviours could change and improve during the interventions. Yet, the literature is scarce in studies aiming to develop training programs to foster the use of teaching motivational strategies among PE teachers taking each specific context into consideration. In this sense, the important role of the individualization of learning has been proven [20,21,22], which promotes perceived competence and self-awareness, as well as considers their own performance and progress according to one’s own pace, which could lead to an enhancement in their teaching behaviours. However, teacher training programs are usually characterized by being massive and generic for all teachers alike, with weak self-regulated learning support or no consideration for their antecedents and current situation [23]. In this sense, the need for lifelong learning programs that allow teachers to evolve and to meet current educational demands has been pointed out [24]. This type of program provides the opportunity to introduce teamwork, which enhances personal and professional development, promotes critical reflections, helps to link theory and practice in a bidirectional way, develops autonomy in teaching and learning or fosters teaching innovation [25,26,27]. However, there is a lack of lifelong learning programs that use the individualization of learning as a driving element that could nurture and improve teaching behaviours.

According to the existing literature and the different theoretical frameworks, the aim of the present study is to design a protocol intervention based on teaching behaviours and motivational strategies grounded in SDT. For this purpose, an individualized teacher training program and intervention will be carried out in the PE context, where teaching motivational strategies will be implemented to foster motivational patterns among students according to the characteristics of each teacher and their specific context of work, which will allow them to adapt their teaching behaviours to their context and, consequently, to improve the quality of teaching.

## 2. Materials and Methods

### 2.1. Design and Participants

A quasi-experimental pre–post study with a mixed-method research approach (mixed methods) will be applied [28,29,30]. Teachers will be involved in a training program on motivational strategies that will be developed during the intervention. To analyse the variables of this study, teachers’ behaviours in PE class will be recorded and analysed through observational analysis before the intervention. Questionnaires will be provided to both teachers and students before and after (pre- and post-test) the intervention. This analysis will be complemented with the interpretation of the teachers’ perceptions during the different meetings, which will be also recorded; a field diary being completed each week; and a focus group discussion at the end of the intervention.

For the video and audio recording, a GoPro Hero8 Black video camera and an audio recorder with a microphone placed on teachers’ clothing will be used, so as not to interfere with their work. Adobe Premiere Pro software will be used to digitalize the records, which show the sessions from the beginning to end by means of a continuous recording from the moment the teacher starts in the practice scenario until the end of the session. Subsequently, teaching behaviours will be coded with Lince Plus [31] qualitative data analysis software.

Teachers will complete pre and post questionnaires via Google Forms before and after the intervention. Following the same procedure, students will fill pre and post questionnaires in the PE class with the presence of a researcher to explain to them the purpose of the project. The study has been agreed upon by the Ethics Committee of the Technical University of Madrid and follows the guidelines of the Declaration of Helsinki and American Psychological Association [32].

**Figure 1:**
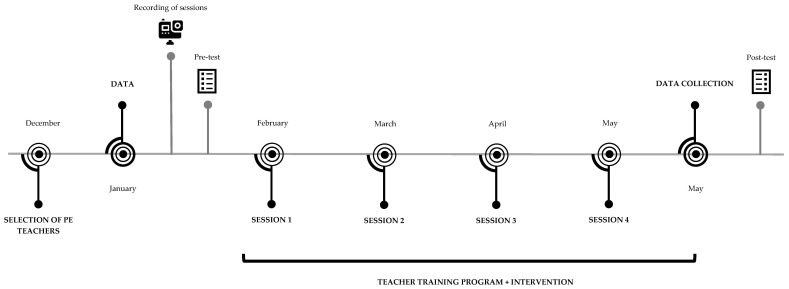
Timeline for data collection and a schematic overview of the study design.

The study will be carried out in Madrid (Spain). Convenience sampling will be conducted in a pre-selection of PE teachers interested in the field. All PE teachers will have similar educational contexts, that is, they teach the subject in high school. In Spain, school courses run from early September to mid-June, including a Christmas break from late December to early January and a break around March or April for the Easter holidays. The intervention will be implemented for five months from January, after the Christmas break, until May (see Figure 1). Assessments will be completed at the beginning of the school course (January) and the end of the intervention (end of May), and a follow-up meeting with teachers will be conducted in the middle of the intervention. It will allow teachers to become familiar with their students from September to December, as well as have enough time to carry out the PE standard assessments during mid-June. The study has been agreed upon by the Ethics Committee of the University and follows the guidelines of the Declaration of Helsinki.

### 2.2. Selection Process and Participant Characteristics

The sample will be accessed through a created database with PE teachers in secondary school who have expressed their interest in engaging in research-related activities or projects. Firstly, teachers will be contacted through an explanatory document with the project’s aims and procedures to take part in the research. The interested teachers will be responsible for sharing the project with principals, as well as for communicating their participation within a maximum of 15 days.

To ensure a blinded process with the participants, the researchers will share with the students that there will be a research project to understand their perceptions of what occurred in PE classes. Parents’ and students’ consent will be required to participate in the study. Convenience sampling will be used to select the class group according with the following inclusion criteria: (I) the teacher must have taught the group class for the whole academic year; (II) the teacher must not have shared teaching with the group; (III) the student has consent to participate; (IV) the student has attended more than 80% of the classes during the intervention; (V) the student has completed the questionnaires of the study variables. The class participating in the study will be randomly selected from among those that meet the selection criteria.

### 2.3. Measures

Observation: A total of two PE classes per teacher will be recorded. To identify the teaching behaviours of both satisfaction and thwarting of autonomy, competence and relatedness towards students, the classification system established by a recent study [11] will be used. Specifically, this classification includes 57 motivational strategies structured according to each BPN satisfaction or frustration: 11 strategies for autonomy satisfaction (e.g., “Allow students’ own-paced progress: allow students to work independently and to solve a problem at their own pace”) and 5 for autonomy thwarting (e.g., “Set pressuring deadlines: allow a capped amount of time for a task, or remind students they are running out of time”); 17 strategies for competence satisfaction (e.g., “Display explicit guidance: provide clear guidance, clear goals, and clear action plans”), and 9 for competence thwarting (e.g., “Undifferentiated challenge: the same task is set for all students regardless of their level of ability”); 7 strategies for relatedness satisfaction (e.g., “Promote cooperation: set up activities that encourage students to work together on tasks”) and 8 for relatedness thwarting (e.g., “Yell or use a harsh tone: teacher yells to get control of the class”).

Teachers’ perceptions of teaching styles: A version adapted to the Spanish context [33] of the Situations-in-School (SIS) questionnaire adapted for the PE context [16] will be used. The scale consists of 12 vignettes of common situations in class. Each situation presents four different reactions, corresponding to one of the four broader teaching styles (autonomy support, structure, control, and chaos) and to one of the eight subareas of teaching styles: participative (e.g., “Offer students a number of different activities that they can choose for the next cycle of education”); attuning (e.g., “Propose exercises that are pleasant, interesting or very attractive”); guiding (e. g., “Give positive feedback, while offering help and advice when necessary”); clarifying (e.g., “Set up a clear and easy-to-follow organization”); demanding (e.g., “Propose a lesson plan for all students to follow. There are no exceptions or excuses”); domineering (e.g., “Firmly insist that “playtime” is over and that now they must show what they are worth”); abandoning (e.g., “They must learn to overcome obstacles on their own”); and awaiting (e.g., “Start the lesson and let it unfold”). Reactions to each situation will be provided on a seven-point Likert scale ranging from 1 (does not describe me at all) to 7 (describes me extremely well).

Students’ perceptions of teaching styles: A version for students adapted to the Spanish context [33] of the Situations-in-School (SIS) questionnaire adapted for the PE context [16] will be used. The scale consists of 12 vignettes of common situations in class. Each situation presents four different reactions, corresponding to one of the four broader teaching styles (autonomy support, structure, control, and chaos) and to one of the eight subareas of teaching styles: participative (e.g., “Offers us a number of different activities that we can choose for the next cycle of education”); attuning (e.g., “Proposes exercises that are pleasant, interesting or very attractive for us”); guiding (e. g., “Gives us positive feedback, while offering us help and advice when we need”); clarifying (e.g., “Sets up a clear and easy-to-follow organization”); demanding (e.g., “Proposes a lesson plan for all of us to follow. There are no exceptions or excuses”); domineering (e.g., “Firmly insists to us that “playtime” is over and that now we must show what we are worth”); abandoning (e.g., “We must learn to overcome obstacles on our own”); and awaiting (e.g., “Starts the lesson and lets it unfold”). Reactions to each situation will be provided on a seven-point Likert scale ranging from 1 (does not describe my teacher at all) to 7 (describes my teacher extremely well).

### 2.4. Teachers’ Training Program and Intervention

PE teachers from participating schools will be involved in a training program on motivational strategies that will be developed during the intervention. Therefore, the implementation of the addressed contents will be carried out by the teachers after the second session. The training program will be structured with a mix of face-to-face and online sessions. The face-to-face sessions will be organized with all the participants together, whereas in online sessions, teachers will be grouped according to their availability. Prior to the first session, the specialist researchers in observational methodology will agree on a date with each teacher to record two PE sessions per teacher in January, as well as to administer the questionnaires to the students of each group in the classroom before the intervention. Additionally, teachers will have to complete the teacher’s questionnaire via Google Forms before attending the first meeting and after the recordings to ensure a blinded process.

The first session of the training program will be held in February and will be face-to-face. The explanation of the project and the theoretical basis will be presented. Firstly, the research team will address basic concepts of SDT in the PE context, and the different teaching styles from the circumplex approach framework. Complementarily, as the contents are presented, practical activities will be carried out in which each teacher will answer in accordance with their teaching experiences. Secondly, a graphic card with the circumplex model will be provided individually for teachers to reflect on their perceptions of their current teaching styles and why they perceive themselves as such. Finally, the results of the circumplex approach variables from the initial questionnaire completed by will be graphically presented. It will allow them to compare their graphic cards with the questionnaire results about their teaching styles’ perceptions.

The second session will take place in March and will be online through videocall. Following the proposal made by the authors [17] to establish a link between the teaching styles and motivational strategies aforementioned, the specialist researchers in observational methodology will select a total of six clips from the two recorded PE classes before the second session. These clips will show teaching situations in which the students’ satisfaction or thwarting of each BPN will be represented. The display of these clips will allow teachers to see themselves from an external point of view, and thus build a self-perception of their teaching. Through these clips, each teacher will identify certain teaching aspects that they would like to change and improve, and how they could implement them in their PE classes.

After this second session, the intervention period will start, in which teachers will have to implement those changes with the selected groups. At the end of each week during the intervention, they will have to complete a field diary through Google Forms, which contains a short questionnaire about their perceptions of the intervention, and three open-ended questions to express successes, problems, or specific comments about the implementation of the improvements.

The third session, also online through videocall, will be held in April. This session will aim to follow up on the intervention of each teacher from a first-person perspective. Therefore, the last theoretical content of the training program about motivational strategies will be presented. In this regard, individually for each teacher, and taking as a reference the changes and improvements that they have proposed in their teaching, a total of 10 motivational strategies from the recent classification system [11] will be selected in accordance with their proposals. The teachers will thus choose three motivational strategies that they would consider feasible to address, specifying in real practice how they would implement each of them.

Finally, the fourth session will take place face-to-face at the end of May. A focus group discussion will be carried out to allow teachers to share their expectations, perceptions and experiences about this individualized training program and intervention, in which each teacher will have personalized their own theoretical content to implement it in the PE class.

### 2.5. Statistical Analysis

The main outcome will be changes and improvements in teaching styles through the perceptions of teachers and students provided by the recorded sessions, interviews and questionnaires. For this purpose, firstly, the nature of the variables will be taken into account through the application of normality, validity and reliability tests.

From a quantitative approach, descriptive statistics (mean and standard deviation) and correlations among the study variables will be calculated to gain an understanding of the teaching styles. Furthermore, given that the aim of this research is to develop an individualized teacher training program and intervention to analyse the effects among teachers and students, in the next phase of the study, in addition to the instruments previously described, motivational regulations, satisfaction and frustration of BPN will be collected from the students. Consequently, a structural equation model will be carried out to analyse the relationship between students’ perceptions of teaching styles, their motivational regulations and the satisfaction and frustration of BPN. From a qualitative perspective, personal interviews and a final focus group will be held to gain information about the potential antecedents of teaching behaviours.

## 3. Discussion

The PE context has been considered an ideal context to promote physical activity habits in students, and it has been proven that the interactions between teachers and students are a key aspect of both teaching and learning behaviours [19,34]. Nevertheless, despite the existence of different studies developing interventions to foster motivation in PE [35], no findings are known so far about PE interventions in motivation in which the teacher personalizes a training program and intervention according to their own characteristics and specific context at work. The aim of the study of the present protocol is for PE teachers to design and apply SDT-based motivational strategies that support autonomy, competence and relatedness in students, and consequently, foster their motivation. From the intervention that has been presented based in the SDT [36], it is expected to make a different contribution to deepen the understanding of interactions in the PE class. Firstly, by developing a replicable teacher-training program where teachers are involved in the design of their own intervention through the implementation of motivational strategies. Secondly, by proposing individualized training that considers teachers in two ways: teachers will be ask what they want to achieve, and what they believe is possible to implement, that is, feasibility perceived by teachers in aspects of their teaching behaviours that could be improved. As a result, it will allow teachers to work on self-awareness, -perception, and -reflection during the intervention. Thirdly, recorded PE sessions will be used to obtain information about their practices and to guide each meeting and interview with the teacher. Finally, the effectiveness of the intervention will be evaluated through both teachers’ and students’ perceptions by asking them specifically about perceptions of teaching styles in PE class.

Regarding the existing literature that has proven the importance of promoting behavioural and motivational outcomes among students [11,19,34], school interventions based on motivational strategies are still scarce [35]. Such studies have addressed teacher training programs in the same way for all the teachers (e.g., [37]), whereas the present study has proposed a personalized training program with an individualized follow-up intervention from the beginning to the end of the process. Besides that, most of these studies have addressed data collection through self-reported measures where participants reflected on their perceptions of the variables analysed (e.g., [38]). Although these assessments tools are useful when presenting adequate values of reliability and validity, the analysis of behaviours through observation has been positioned as an interesting alternative [39] that allows a deepening of the knowledge of the interactions that occur [40]. What is more, the combinations of both ways to collect data would be an interesting approach. On this basis, the present study will use mixed methods through both qualitative and quantitative methodologies to enrich the analysis by comparing perceptions of behaviours and real-life teaching in PE classes.

Based on the previous literature addressed, expected outcomes of the intervention could be pointed out in relation to the aim of the present study. First of all, the training program is expected to enhance self-awareness, -perception and -reflection on teaching practices among teachers, which could improve their perceptions of competence in the work context. It is also worth highlighting that the main feature of this training program is the personalization, where teachers design their implementation in the PE class, and individualization, where teachers have an individual follow-up during the whole intervention. As a result of these findings, it is expected that the intervention program will encourage teachers to change and improve their teaching behaviours by implementing motivational strategies that support students’ needs, and consequently, to modify their teaching styles in the long term.

The present study shows some limitations that are worth noting. The first limitation is related to the duration of the intervention. The training program could be extended to one or two more sessions to address the contents in more depth and gradually. These sessions could be developed at the beginning of the academic year, in the second month, in October, to allow teachers to meet the class groups and to begin developing the PE contents. Similarly, a longer intervention will facilitate the assimilation and internalization of the changes and improvements about teaching behaviours that teachers will implement in their PE classes. A second limitation is related to the follow-up process during the intervention, in which teachers are required to complete a field diary each week and attend monthly meetings. The need to have a supervisor in order to check and remember the diary each week could lead to a perception of “must-do” and overload among teachers that may cause them to complete it more superficially and randomly, or just do not complete it at all. A third limitation refers to the measures used, in which only perceptions of teaching styles from both teachers’ and students’ point of view will be assessed. It would be interesting to measure different motivational outcomes to analyse how these variables interact with teaching styles.

## 4. Conclusions

The present study aims to design a protocol intervention based on teaching behaviours and motivational strategies grounded in SDT. Through the proposal of an individualized training program, it is expected that teachers will be able to change their teaching styles through the implementation of motivational strategies that will allow them to improve their teaching patterns. Consequently, the use of these motivational strategies would promote behavioural and motivational outcomes among students.

This study represents the first attempt from an individualized training program in which teachers could personalize their teaching interventions according to their specific contexts. This study would increase the understanding of the interplay between the different perceptions of teachers and students, and compare it with actual practice in PE classes. Through the analysis of both quantitative—using questionnaires—and qualitative data—using observations and the information provided in the field diary and meetings—the results will allow us to deepen the understanding of the interactions between teachers and students in PE class, and in turn, improve teaching quality through the promotion of motivation.

## Data Availability

Not applicable.
